# Supplementary material for: Widespread low-affinity motifs enhance chromatin accessibility and regulatory potential in mESCs
Source: bioRxiv. 2025 Nov 19:2025.11.18.685822. Preprint. [Version 1] doi: 10.1101/2025.11.18.685822 (PMC12667796; doi:10.1101/2025.11.18.685822)
Supplement: Supplement 2 [file NIHPP2025.11.18.685822v1-supplement-2.pdf]

bioRxiv preprint doi: https://doi.org/10.1101/2025.11.18.685822; this version posted November 19, 2025. The copyright holder for this preprint (which was not certified by peer review) is the author/funder, who has granted bioRxiv a license to display the preprint in perpetuity. It is made available under aCC-BY 4.0 International license.

## Supplemental figures

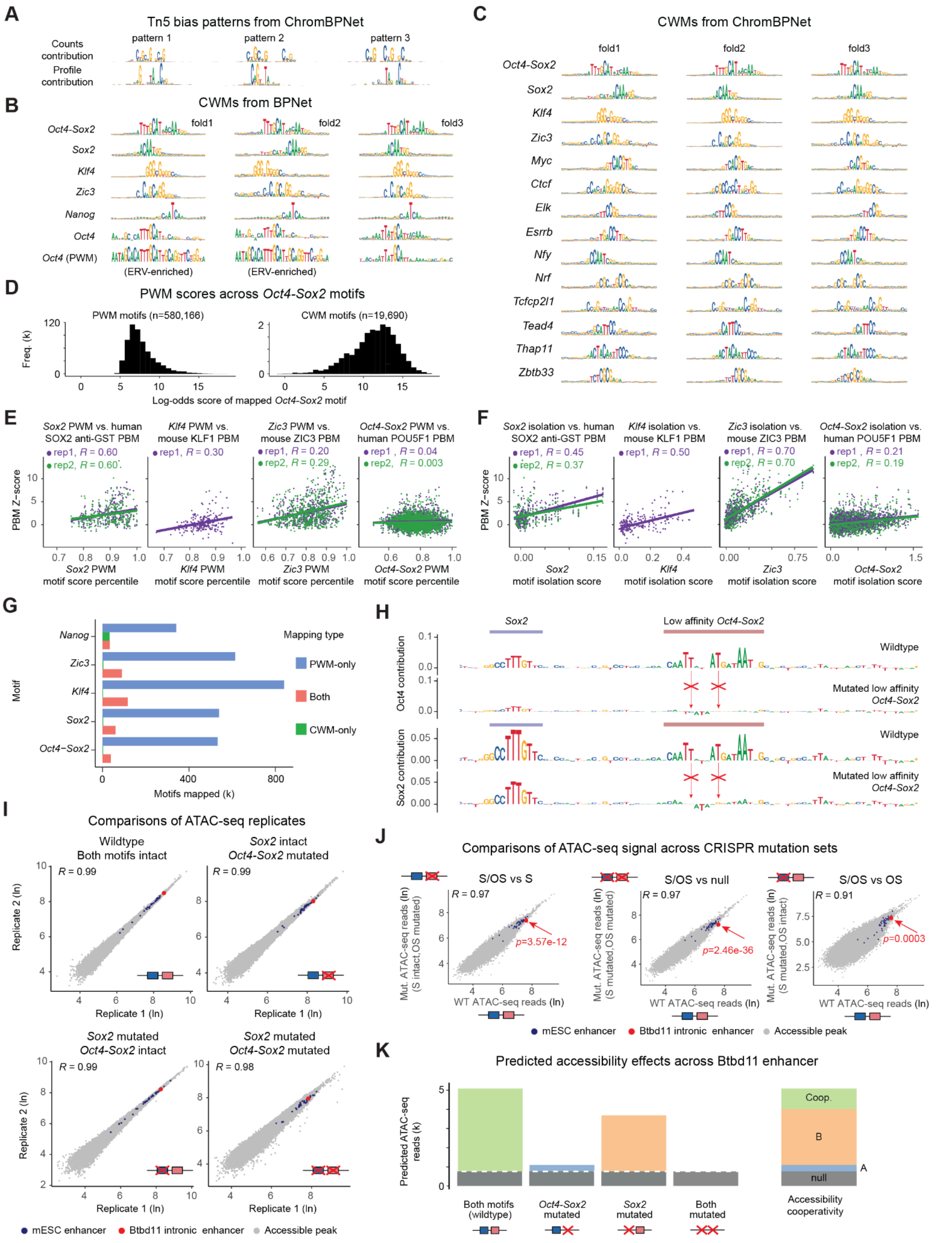


**Supplemental Figure 1**

**A)** The ChromBPNet Tn5 bias model learned expected bias motifs, shown as PWMs for the top three patterns, summarized by TF-MoDISco from sequences with high counts contribution or profile contribution scores.

**B-C)** Motifs shown as CWMs learned by **(B)** the BPNet binding model and **(C)** the ChromBPNet accessibility model across different folds as summarized by TF-MoDISco. While most motifs were robustly discovered in all folds, the single *Oct4* motif was not robustly discovered, often returning ERV-enriched mappings as these are recognizable in the *Oct4* PWMs (bottom row).

**D)** Log-odds scores of *Oct4-Sox2* motifs learned by the accessibility model (right, n=19,960) show a broader distribution of affinity scores compared to *Oct4-Sox2* motifs mapped using PWM scanning (left, n=580,166).

**E-F)** Scatterplots showing a relationship between model-derived motif features and available *in vitro* measured PBM binding data for human SOX2, mouse KLF1, mouse ZIC3 and human POU5F1 (OCT4), the TFs in the UniPROBE's database that best match our TFs of interest. The PBM binding data are summarized as Z-scores for the 8-mer sequences that match the *Sox2*, *Klf4*, *Zic3* and *Oct4-Sox2* motifs. **(E)** The PWM score percentiles of all uniquely mapped motif sequences and **(F)** the corresponding binding isolation scores both correlate with the PBM Z-scores, as shown by Spearman correlation scores. PBM replicate experiments are shown in separate colors.

**G)** Comparison of the number of motifs discovered only by PWM scanning, only by CWM scanning or both, using a low-stringency cutoff for PWM scanning necessary for discovering low-affinity motifs. As expected, PWM scanning discovered vastly more motifs. Motifs discovered by CWM scanning were typically a subset of the motifs discovered by PWM scanning, except for *Nanog* motifs, which are more difficult to map with PWM scanning.

**H)** The intronic *Btbd11* enhancer with contribution scores from the BPNet binding model for Oct4 and Sox2 across the wildtype sequence (top) and mutated sequence (bottom). This shows that mutating the low-affinity *Oct4-Sox2* motif abolishes the predicted binding of Oct4 and Sox2. Note that the *Sox2* motif on the left is still predicted to be bound, although with slightly lower contribution in the mutant.

**I)** ATAC-seq replicate comparisons show that the ATAC-seq experiments on wildtype and CRISPR clones mutating the *Btbd11* enhancer are reproducible (Spearman correlation coefficients reported on the scatterplots). Total reads were measured across ATAC-seq peaks found in wildtype experiments.

**J)** Direct comparisons of ATAC-seq reads between wildtype and CRISPR *Btbd11* mutant experiments. As expected, the Spearman correlation coefficients are overall high but show differential accessibility across the *Btbd11* region, as calculated by DESeq2 with adjusted p values (red arrows).

**K)** Accessibility across the *Btbd11* region as predicted by the ChromBPNet model for wildtype and after the *Sox2* and/or low-affinity *Oct4-Sox2* motif sequences were mutated. Changes were measured across coordinates chr10:85539400-85539800. To the right, predicted cooperative effects (green) are stacked upon individual marginal effects of motifs.

bioRxiv preprint doi: https://doi.org/10.1101/2025.11.18.685822; this version posted November 19, 2025. The copyright holder for this preprint (which was not certified by peer review) is the author/funder, who has granted bioRxiv a license to display the preprint in perpetuity. It is made available under aCC-BY 4.0 International license.

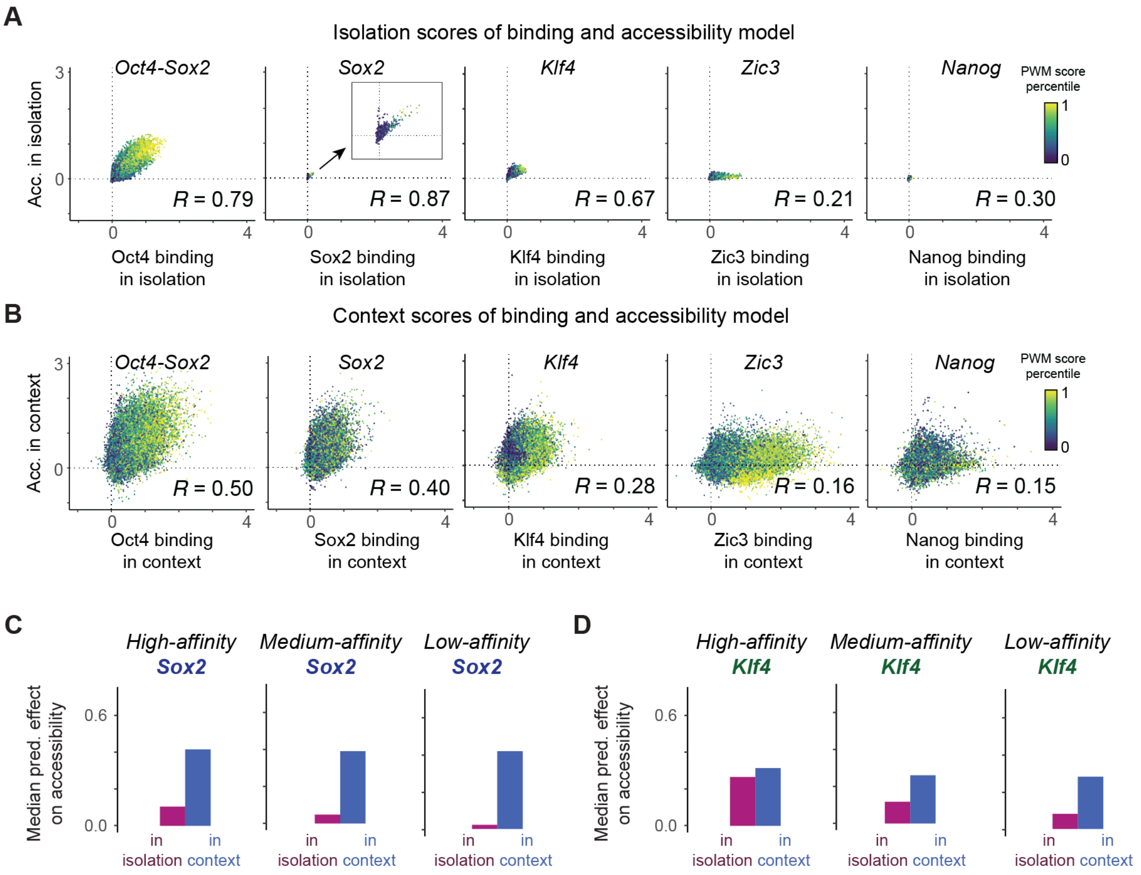


**Supplemental Figure 2**

**A-B)** Comparison of the **(A)** isolation scores or **(B)** context scores obtained from the binding model (x-axis) and the accessibility model (y-axis) for the *Oct4-Sox2*, *Sox2*, *Klf4, Zic3* and *Nanog* mapped motifs, colored by their PWM score percentiles.

**C-D)** For all sets of motifs motifs mapped by only accessibility models, median isolation and context scores predicted by the accessibility model for **(C)** *Sox2* motifs and **(D)** *Klf4* motifs of high, medium and low affinity motifs (5k each, based on PWM score) show that low-affinity motifs receive a bigger boost in context than high-affinity motifs.

bioRxiv preprint doi: https://doi.org/10.1101/2025.11.18.685822; this version posted November 19, 2025. The copyright holder for this preprint (which was not certified by peer review) is the author/funder, who has granted bioRxiv a license to display the preprint in perpetuity. It is made available under aCC-BY 4.0 International license.

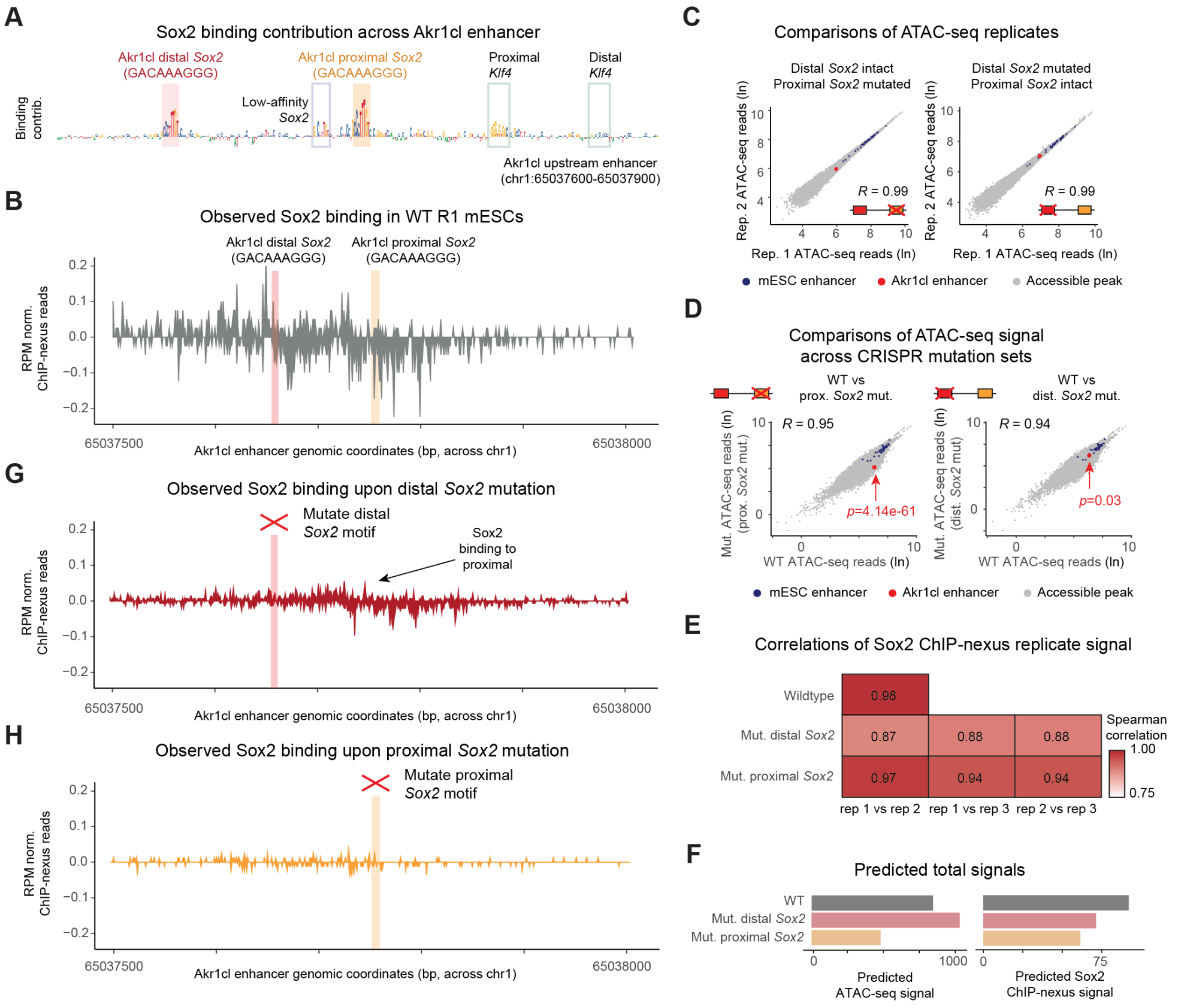


**Supplemental Figure 3**

**A)** Sox2 binding contribution scores of the Akr1cl upstream enhancer (chr1: 65037600-65037900) with contributing motifs marked.

**B)** RPM-normalized Sox2 ChIP-nexus across the Akr1cl enhancer, denoting distinct Sox2 binding profiles at both *Sox2* motifs.

**C)** Replicate comparisons measuring total ATAC-seq reads across reproducible ATAC-seq peaks from wildtype and CRISPR *Akr1cl* mutant experiments. Spearman correlation coefficients are reported on scatterplots.

**D)** Pairwise comparisons of pooled ATAC-seq reads from wildtype and CRISPR Akr1cl mutant experiments. Spearman correlation coefficients are reported on scatterplots. Differential accessibility between wildtype and each CRISPR Akr1cl mutant experiment was calculated using DESeq2, with adjusted significance values reported for the Akr1cl enhancer (red arrows).

**E)** Replicate Spearman correlations measuring total Sox2 ChIP-nexus reads across reproducible ATAC-seq peaks from CRISPR *Akr1cl* mutant experiments.

**F)** Predicted ATAC-seq signal or predicted Sox2 ChIP-nexus signal occurring across chr1: 65037600-65037900 across wildtype (black), distal *Sox2* mutant (red) and proximal *Sox2* mutant (orange) sequences.

**G-H)** RPM-normalized Sox2 ChIP-nexus profiles across the *Akr1cl* enhancer from **(G)** the distal *Sox2* mutation and the **(H)** the proximal *Sox2* mutation CRISPR experiments.

bioRxiv preprint doi: https://doi.org/10.1101/2025.11.18.685822; this version posted November 19, 2025. The copyright holder for this preprint (which was not certified by peer review) is the author/funder, who has granted bioRxiv a license to display the preprint in perpetuity. It is made available under aCC-BY 4.0 International license.

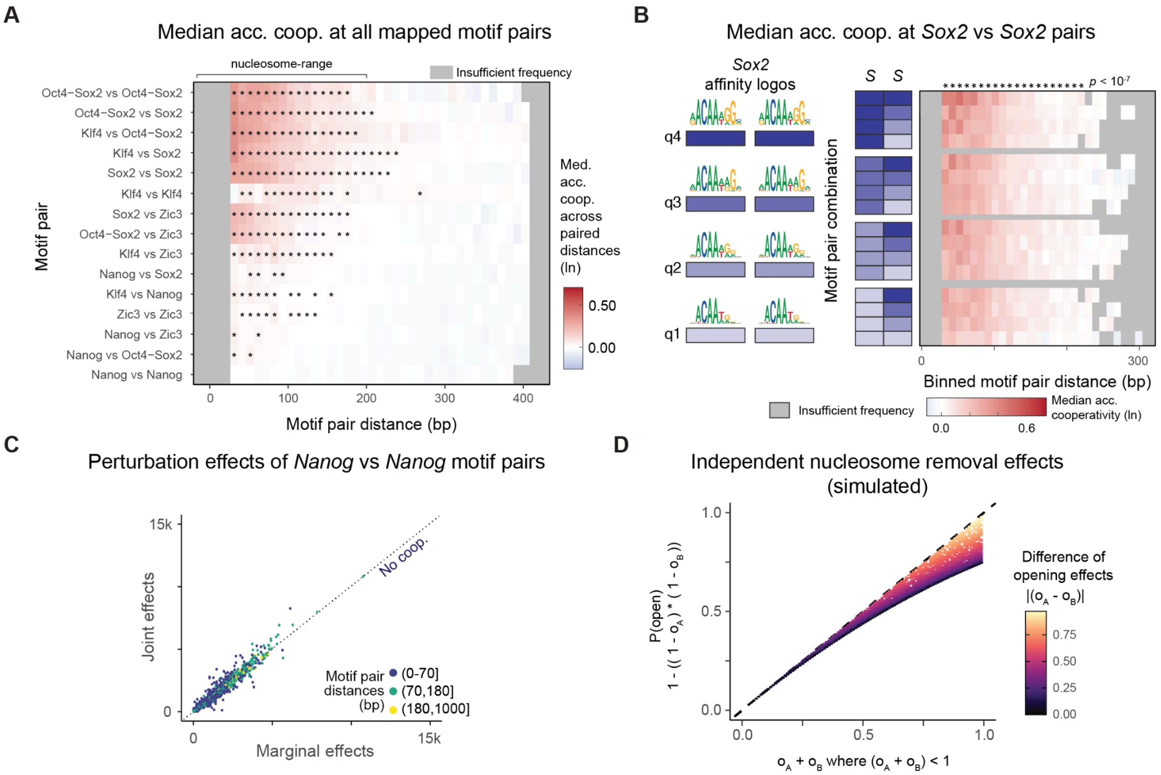


**Supplemental Figure 4**

**A)** Median accessibility cooperativity across all motif pairs of key mESC motifs, separated by motif pair distances. Motif/distance combinations with fewer than 20 motif pairs were not considered (gray boxes). Cooperativity significance was derived from a one-tailed Wilcoxon test comparing whether motif pairs arranged at select binned distances were more cooperative than motif pairs arranged at very long distances (>400 bp) with multiple-comparison Bonferroni corrections and an adjusted significance cutoff of $p < 10^{-7}$.

**B)** Median accessibility cooperativity between *Sox2*/*Sox2* motif pairs across quartiles of motif affinity and motif pair distances, binned to 10 bp. Motif/distance combinations with fewer than 20 motif pairs were not considered (gray boxes). Cooperativity significance was derived from a one-tailed Wilcoxon test comparing whether *Sox2*/*Sox2* motif pairs arranged at select binned distances were more cooperative than *Sox2*/*Sox2* motif pairs arranged at very long distances (>400 bp) with multiple-comparison Bonferroni corrections and an adjusted significance cutoff of $p < 10e^{-7}$.

**C)** Comparison of joint and marginal predicted accessibility effects for the non-pioneer *Nanog*/*Nanog* motif pairs, colored by the pairs' relative center-to-center distances to one another.

**D)** Opening likelihoods for two pioneer motifs ($o_A$ = [0.01, 1] and $o_B$ = [0.01, 1] where $o_A + o_B < 1$) were independently sampled (n=10,000) and simulated based on the premise that each pioneer TF independently removes the same nucleosome with a certain probability ($P(open) = 1 - (( 1 - o_A ) * ( 1 - o_A )))$, producing less than additive effects.

bioRxiv preprint doi: https://doi.org/10.1101/2025.11.18.685822; this version posted November 19, 2025. The copyright holder for this preprint (which was not certified by peer review) is the author/funder, who has granted bioRxiv a license to display the preprint in perpetuity. It is made available under aCC-BY 4.0 International license.

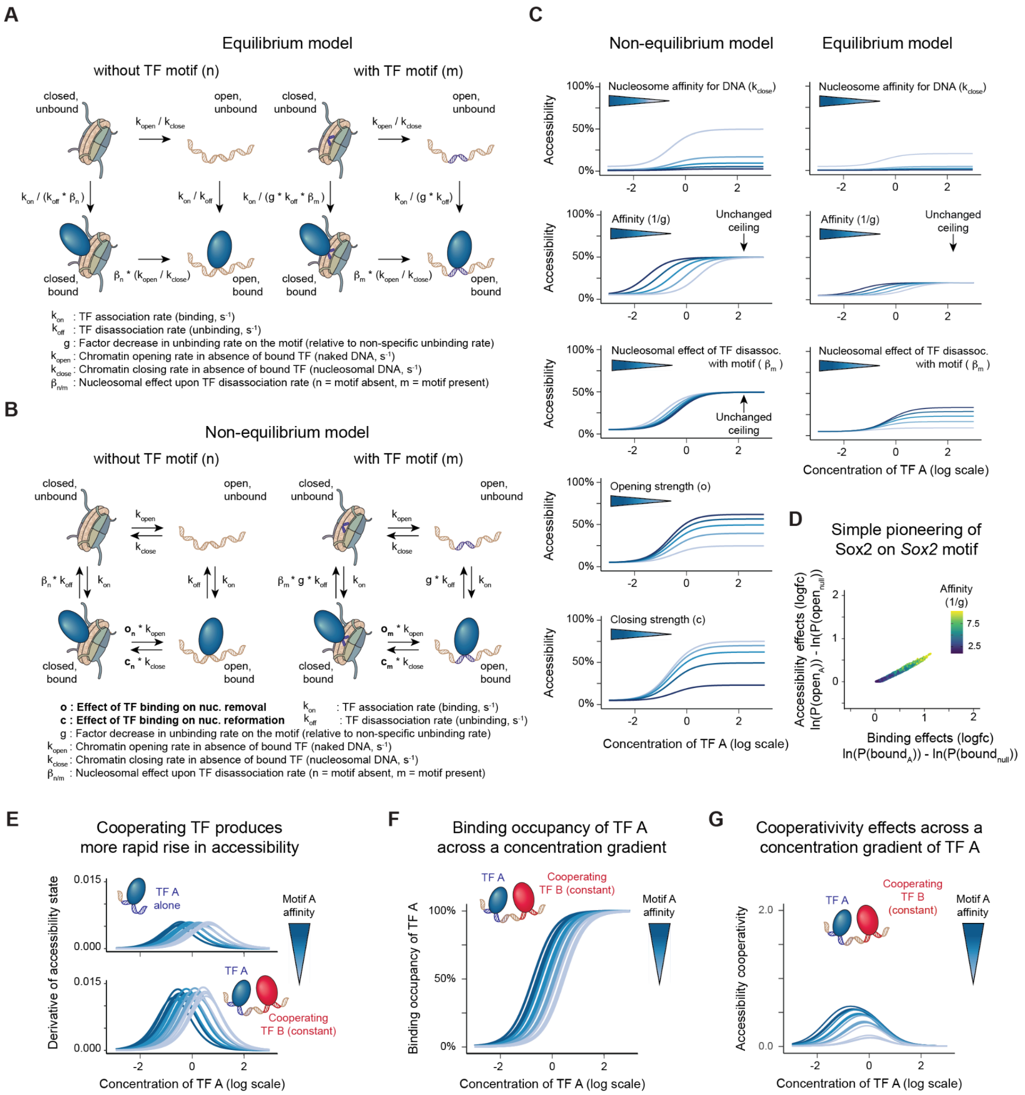


**Supplemental Figure 5**

**A)** Parameterized graphic depicting a kinetic model in equilibrium of bound TF (called A) effects on nucleosome removal and reformation. Transitions are reversible, but they are only shown in one direction given that the equilibrium steady-state is only determined by the ratios between forward and backward rates, as indicated. On the left, the motif is not present and therefore recruits only non-specific binding to the TF. On the right, the motif is present and recruits specific TF binding, with motif affinity effectively given by the factor g.

**B)** Parameterized graphic depicting a kinetic model out of equilibrium of bound TF effects on nucleosome removal and reformation. Parameter definitions are the same from graphical representation in **(A)**. Additional non-equilibrium parameters include o (effect of bound TF on nucleosome removal) and c (effect of bound TF on nucleosome reformation). Note that a higher value of c means less of an effect).

**C)** 1 TF kinetic model simulations (n=1) in equilibrium (left) and non-equilibrium (right) showing the accessibility state over changing concentrations for a range of changing model parameters. We tested ranges of nucleosome intrinsic affinity for DNA, motif affinity, nucleosome effect upon TF disassociation in the presence of a motif, and in the case of the non-equilibrium model, TF binding effects on nucleosome removal and reformation. For the equilibrium model, the parameters were set to simulate moderate pioneering in the presence of a nucleosome with low intrinsic affinity to DNA. Parameter ranges can be found in Table S5 (equilibrium model) and Table S6 (non-equilibrium model).

**D)** 1 TF kinetic model (out of equilibrium) simulations (n=50,000) showing the correlation between accessibility state, binding occupancy and motif affinity in the presence of a pioneer TF. Parameter ranges can be found in Table S6. Note that $c = 1$ (effect of TF on nucleosome reformation), indicating that the simulated simple pioneering does not allow reduction of nucleosome reformation as a mechanism to increase accessibility, only nucleosome removal.

**E)** Derivatives of accessibility curves from the 1 TF kinetic model (Figure 5B) and the 2 TF kinetic model (Figure 5E) showing that the cooperative (2 TF) conformation produces a more rapid rise in accessibility.

**F)** 2 TF kinetic model (out of equilibrium) simulations (n=5) measuring binding occupancy of TF A over a range of TF A motif affinities across changing concentrations of TF A. TF B affinity and concentration was kept constant. As concentration of TF A increases, binding occupancy reaches the maximum value of 100%, unlike accessibility state which plateaus at values less than 100% (Figure 5E). Parameter ranges can be found in Table S7.

**G)** 2 TF kinetic model (out of equilibrium) simulations (n=5) measuring accessibility cooperativity between TF A and TF B over a range of TF A motif affinities across changing concentrations of TF A. Though TF B affinity and concentration was kept constant, the presence of B caused accessibility states to be cooperatively enhanced. Note that the cooperativity disappears at high TF A concentrations and the maximum cooperativity is increased with affinity. Parameter ranges can be found in Table S7.

bioRxiv preprint doi: https://doi.org/10.1101/2025.11.18.685822; this version posted November 19, 2025. The copyright holder for this preprint (which was not certified by peer review) is the author/funder, who has granted bioRxiv a license to display the preprint in perpetuity. It is made available under aCC-BY 4.0 International license.

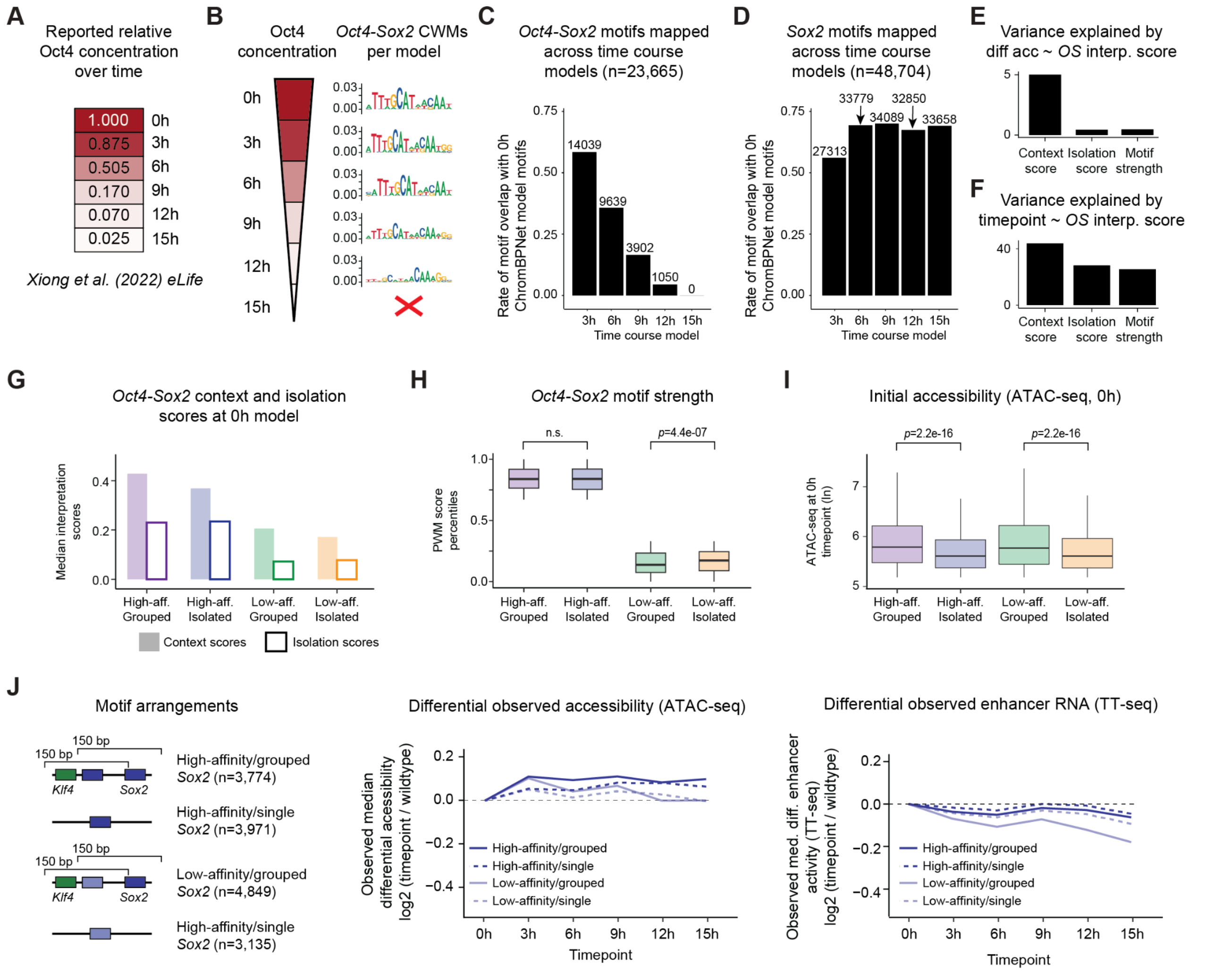


**Supplemental Figure 6**

**A)** Reported relative rates of Oct4 upon doxycycline withdrawal time course.

**B)** Accessibility TF-MoDISco CWMs of discovered *Oct4-Sox2* motifs from accessibility models of all Oct4 concentration timepoints. Accessibility model capturing the 15h time point failed to discover a contributing role of the *Oct4-Sox2* motif to accessibility.

**C-D)** Rates by which **(C)** *Oct4-Sox2* and **(D)** *Sox2* motifs mapped by the reference 0h timepoint model were mapped by the corresponding time course accessibility models. Overall mapping frequencies are reported above each rate.

**E)** Barplots measuring the variance explained from interpretation scores to explain *Oct4-Sox2* motif (mapped by the 0h timepoint) effects on observed differential accessibility changes over the concentration time course using independent linear regression.

**F)** Barplots measuring the variance explained from interpretation scores to explain *Oct4-Sox2* motif (mapped by the 0h timepoint) effects on which timepoint (treated as a numeric value) each *Oct4-Sox2* motif was last mapped across using independent linear regression for each interpretation method.

**G)** Median context and isolation scores of *Oct4-Sox2* motifs mapped by the 0h timepoint model. While both grouped and single motif arrangements possess higher context scores than isolation scores (likely because grouped arrangements only consider *Sox2* and *Klf4* motif partners and not other motifs), grouped arrangements possess a higher relative ratio of the two interpretation scores.

**H)** Boxplots depicting PWM score percentiles of *Oct4-Sox2* motifs across the motif arrangements denoted in Figure 6E. Statistics were performed using a Wilcoxson test.

**I)** Boxplots depicting initial ATAC-seq reads at the 0h timepoint across the motif arrangements denoted in Figure 6E. Statistics were performed using a Wilcoxson test. Note that grouped motif arrangements possess intrinsically higher initial accessibility levels than single motif arrangements.

**J)** Graphic (left) denoting arrangements of single/grouped and strong/weak *Sox2* motifs without an *Oct4-Sox2* motif nearby. Median experimental differential accessibility levels (middle) measured over the concentration time course across regions with either single (solid line) or grouped (dashed line) high- and low-affinity *Sox2* motifs. Median experimental differential enhancer expression levels (right) measured over the concentration time course using TT-seq across regions with either single (solid line) or grouped (dashed line) high- and low-affinity *Sox2* motifs. *Sox2* motifs were derived from the 0h timepoint model, while high- and low-affinity motifs were the top and bottom tertiles of PWM scores.
